# Effects of Intra-articular Bone Marrow Aspirate Infiltration in the Treatment of Knee Osteoarthritis: A Clinical Study Comparing BMA versus Corticosteroid and Genicular Block

**DOI:** 10.1055/s-0044-1800942

**Published:** 2025-06-14

**Authors:** Renata Clazzer, Dilamar Moreira Pinto, Mariana Valois de Aquino Krause, Tale Lucas Vieira Rolim, Ricardo Lyra de Oliveira, Diego Ariel de Lima

**Affiliations:** 1 Hospital Otavio de Freitas, Recife, PE, Brazil.; 2 Health Sciences Department, Universidade Federal Rural do Semi-Árido, Mossoró, RN, Brazil.

**Keywords:** bone marrow, injections, intra-articular, osteoarthritis, knee, pain management, injeções intra-articulares, manejo da dor, medula óssea, osteoartrite de joelho

## Abstract

**Objective** To assess the efficacy of autologous bone marrow aspirate (BMA) in reducing pain and improving functionality in patients with knee osteoarthritis (OA), in comparison with intraarticular corticosteroid injection and genicular nerve block.

**Methods** A prospective, randomized, controlled, single-blinded, comparative, and analytical clinical study was conducted. There were 50 patients with knee OA divided into two groups: an intervention group receiving BMA treatment and a control group undergoing standard corticosteroid articular infiltration and genicular block. Outcome measures were evaluated using the Western Ontario and McMaster universities osteoarthritis index (WOMAC).

**Results** After 6 months, significant pain reduction was noted in the BMA group compared with the control group ( *p* = 0.030). No significant differences were found in stiffness and physical activity scores between the groups. The intervention group demonstrated significant improvements in all assessed WOMAC subcategories pre- and posttreatment.

**Conclusions** Treatment with BMA can significantly reduce pain, potentially leading to an improved functionality, suggesting its potential as a viable therapeutic option for managing knee OA.

## Resumo

**Objetivo** Avaliar a eficácia do aspirado de medula óssea autólogo (BMA) na redução da dor e melhoria da funcionalidade em pacientes com osteoartrite (OA) de joelho, comparando com a infiltração intra-articular com corticoide e bloqueio genicular.

**Métodos** Foi conduzido um estudo clínico prospectivo, randomizado, controlado, simples-cego, comparativo e analítico. Foram 50 pacientes com osteoartrite de joelho, divididos em dois grupos: um grupo de intervenção recebendo tratamento com BMA e um grupo controle submetido à infiltração articular padrão com corticoide e bloqueio genicular. Os resultados foram avaliados usando o Índice de Osteoartrite das Universidades de Western Ontario e McMaster (WOMAC).

**Resultados** Após 6 meses, uma redução significativa da dor foi observada no grupo BMA em comparação ao grupo controle ( *p* = 0.030). Não foram encontradas diferenças significativas nos escores de rigidez e atividade física entre os grupos. O grupo de intervenção demonstrou melhorias significativas em todas as subcategorias do WOMAC avaliadas antes e após o tratamento.

**Conclusão** O tratamento com BMA pode reduzir significativamente a dor, possibilitando uma possível melhora funcional, sugerindo seu potencial como uma opção terapêutica viável no manejo da OA de joelho.

## Introduction

Knee osteoarthritis (OA) is a chronic degenerative condition predominantly affecting females and resulting in the progressive deterioration of the articular cartilage. This disease leads to deformities in the joint, along with possible muscular and ligamentous imbalances, particularly in areas subjected to greater load, as evidenced by typical radiographic features including bone sclerosis, cysts, and osteophytes. 1 2 3

The presence of knee OA significantly impacts physical performance and is considered one of the top ten causes of disability globally. Conservative therapeutic approaches commonly employed for the treatment of this condition include weight loss, physical exercises, administration of nonsteroidal anti-inflammatory drugs (NSAIDs), analgesics, intraarticular injections containing hyaluronic acid (HA) and glucocorticoids, and genicular blocks. 4

Recently, orthobiologic injections have emerged as a potentially safe and effective option for treating knee OA, including bone marrow aspirate (BMA), mesenchymal stem cells (MSCs), and platelet-rich plasma (PRP). 5 The use of BMA as an innovative cell therapy stands out because its technique is simple, presents low morbidity, and provides MSCs. These cells have the ability to promote the repair of articular tissue and influence the expression of cytokines IL-8 and -1β, and serve as a source of intracellular signaling peptides, such as platelet-derived growth factor (PDGF), transforming growth factor-β (TGF-β), and vascular endothelial growth factor (VEGF). 6 7 Thus, orthobiologic injections can be an excellent option in the treatment of gonarthrosis.

Therefore, the aim of this study is to evaluate the efficacy of using autologous BMA to reduce pain and improve functionality in patients with knee OA, in comparison with intra-articular corticosteroid injection and genicular nerve block.

## Materials and Methods

Following approval by the Research Ethics Committee (CAAE: 1164923.6.0000.5200), a prospective, randomized, controlled, longitudinal, single-blind (evaluators), comparative, descriptive, and analytical clinical study was conducted. This study involved patients with knee OA who were treated at the orthopedics service of our institution. The patients were organized into two groups through block randomization, with the only specification being an equivalent number of patients in each group. Group 1 (intervention) was assigned to receive treatment with BMA; Group 2 (control) was assigned to receive treatment with articular corticosteroid infiltration and genicular block, standard at the institution. All procedures were performed by the same surgeon.

The number of sample components was stipulated to ensure a 95% confidence interval (CI), a power of approximately 80%, and a between-group difference of 20%; that is, 25 people in the intervention group and 25 in the control group, totaling 50 individuals. 8

Inclusion criteria were patients between 30 and 90-years-old, with OA grades II to IV according to the Kellgren and Lawrence scale, 9 absence of other inflammatory rheumatic diseases, no prior treatment with corticosteroids, either injectable or oral, in the past 12 months, and who signed the informed consent form.

Exclusion criteria were those with any condition that precluded follow-up, loss of follow-up/contact with the patient, use of oral or IV corticosteroids during the follow-up period, hemoglobin less than 11 g/dl, platelet count less than 150,000/mm ^3^ , or any coagulation disorder.

### Group 1 (Intervention): BMA Treatment

**Fig. 1:**
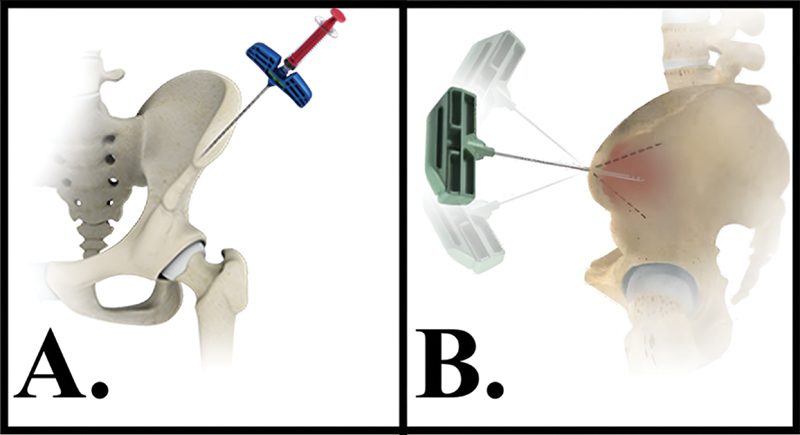
Bone marrow aspirate (BMA). ( **A** ) Puncture at the iliac crest near the anterior superior iliac spine, with a 40° inclination from lateral to medial and 40°o from superior to inferior. ( **B** ) The needle can be withdrawn and reinserted with a slight change in angulation to aspirate bone marrow from another site, which may improve the concentration of MSCs.

**Fig. 2:**
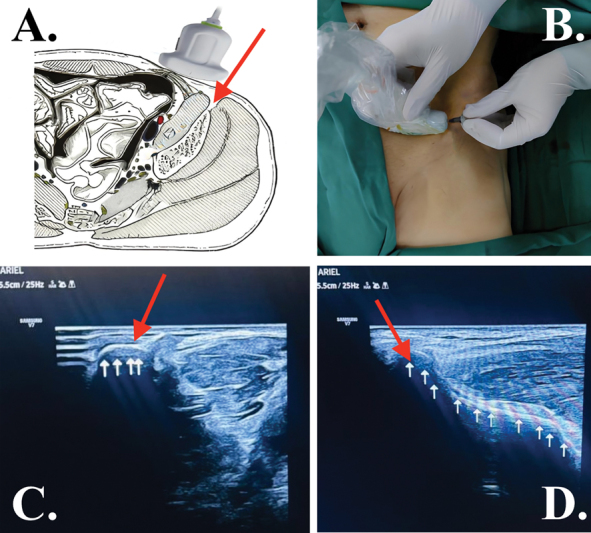
Bone marrow aspirate (BMA). ( **A** ) Schematic figure showing the ultrasound probe transversely aligned with the iliac bone. ( **B** ) Patient image showing the ultrasound probe transversely aligned with the iliac bone. ( **C** ) Ultrasound image corresponding to the position of the ultrasound probe transversely aligned with the iliac bone. ( **D** ) By rotating the probe 90°, the ultrasound image corresponding to the position of the ultrasound probe longitudinally aligned with the iliac bone. Red arrow, Iliac puncture site; white arrows, iliac cortical bone.

Using ultrasound guidance, approximately 15 ml of BMA was drawn from the anterosuperior iliac spine into heparinized syringes, using a multi-site low-volume technique, 10 as shown in Figs. 1 and 2 . The patient, under sedation and local anesthesia with 1% lidocaine, BMA was collected using an 11 G biopsy needle/canula.

**Fig. 3:**
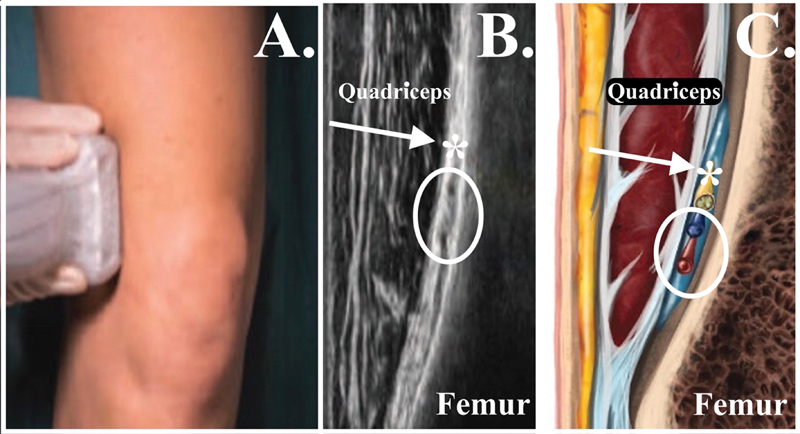
Anatomical landmarks for the lateral superior genicular nerve (LSGN) block. ( **A** ) Transducer in the coronal plane at the distal lateral metaphyseal region of the femur. ( **B** ) Ultrasound image obtained with the transducer in the coronal plane at the distal lateral metaphyseal region of the femur. ( **C** ) Schematic image in the coronal plane at the distal lateral metaphyseal region of the femur. White arrow and *, lateral superior genicular nerve; white circle, lateral superior genicular vein and artery.

**Fig. 4:**
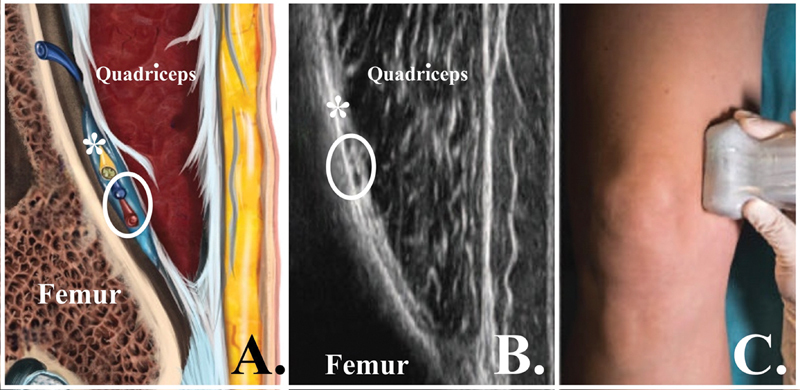
Anatomical landmarks for the medial superior genicular nerve (MSGN) block. ( **A** ) Schematic image in the coronal plane at the distal medial metaphyseal region of the femur. ( **B** ) Ultrasound image obtained with the transducer in the coronal plane at the distal medial metaphyseal region of the femur. ( **C** ) Transducer in the coronal plane at the distal medial metaphyseal region of the femur. *Medial superior genicular nerve; white circle, medial superior genicular vein and artery.

**Fig. 5:**
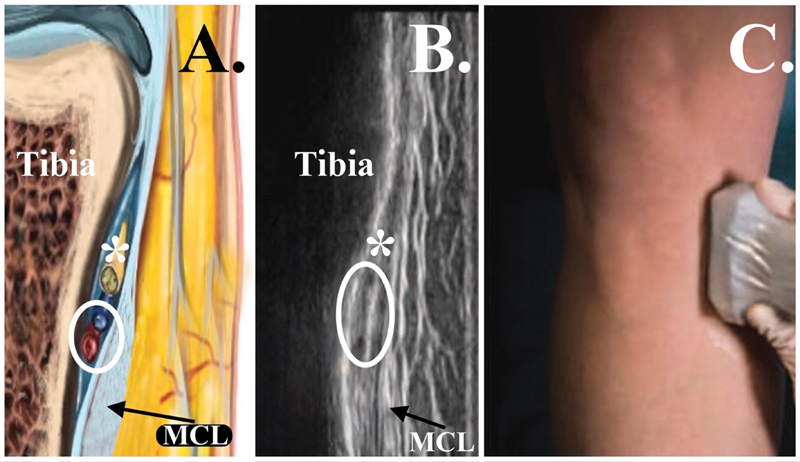
Anatomical landmarks for the medial inferior genicular nerve (MIGN) block. ( **A** ) Schematic image in the coronal plane at the proximal medial metaphyseal region of the tibia. ( **B** ) Ultrasound image obtained with the transducer in the coronal plane at the proximal medial metaphyseal region of the tibia. ( **C** ) Transducer in the coronal plane at the proximal medial metaphyseal region of the tibia. *Medial inferior genicular nerve; white circle, medial inferior genicular vein and artery. Abbreviation: MCL, medial collateral ligament.

**Fig. 6:**
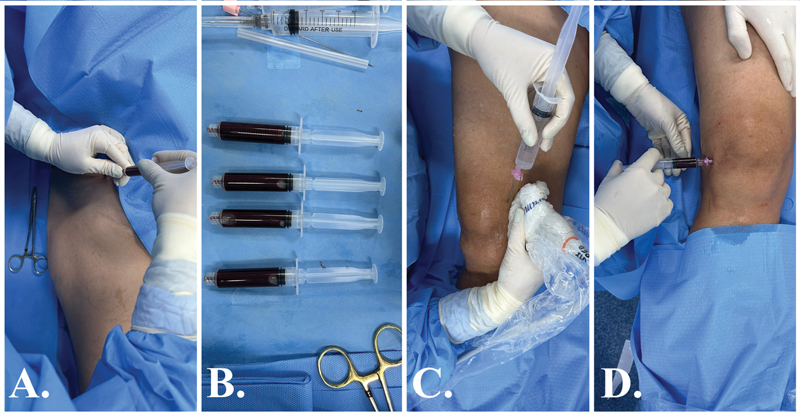
Group 1 (Intervention), BMA treatment. ( **A** ) BMA from the anterosuperior iliac spine using heparinized syringes. ( **B** ) Approximately 15 ml of bone marrow. ( **C** ) A 20 ml solution was prepared consisting of 8 ml of ropivacaine (10 mg/ml), 10 ml of 50% dextrose, and 2 ml of dexamethasone (4 mg/2.5 ml). This solution was used for genicular branch blocks, guided by ultrasound. From it, 5 ml each were applied to the medial femoral, lateral femoral, and medial tibial genicular branches. ( **D** ) The remaining 5 ml of the previous solution (ropivacaine + dextrose + dexamethasone) were mixed with the 15 ml of BMA and intraarticularly infiltrated into the knee in question.

A 20 ml solution was prepared consisting of 8 ml of ropivacaine (10 mg/ml), 10 ml of 50% dextrose, plus 2 ml of dexamethasone (4 mg/2.5 ml). This solution was used for genicular branch blocks, guided by ultrasound. 11 Then, 5 ml was injected into the medial femoral, 5 ml into the lateral femoral, and 5 ml into the medial tibial genicular branches ( Figs. 3 , 4 and 5 ). The remaining 5 ml were mixed with the 15 ml of BMA and intraarticularly infiltrated into the knee in question ( Fig. 6 ).

### Group 2 (Control): Articular Corticosteroid Infiltration and Genicular Block, Standard of the Institution

A 20 ml solution was produced consisting of 8 ml of ropivacaine (10 mg/ml), 10 ml of 50% dextrose, plus 2 ml of dexamethasone (4 mg/2.5 ml). This solution was used for the genicular branch blocks, guided by ultrasound. Then, 5 ml was injected into the medial femoral, 5 ml into the lateral femoral, 5 ml into the medial tibial genicular branches. The remaining 5 ml were to be intraarticularly infiltrated into the knee in question. This procedure is standard at our institution and the only difference compared with Group 1 is that the control group does not receive BMA infiltration.

### Patient Evaluation

All patients were encouraged to discontinue the use of NSAIDs 2 weeks before and several weeks after treatment. If patients experienced postprocedural pain, rescue medication with opioids was prescribed for up to 5 days. Patients were advised to avoid activities that could exacerbate pain throughout their rehabilitation protocol, which would begin with rest and home/community ambulation. The progression of physical activities included swimming or low-impact exercise, followed by walking, resistance training, running, and finally advancing to full functional activity.

The variables analyzed in each group were age, gender, and laterality. To evaluate the therapeutic response, the Western Ontario and McMaster universities osteoarthritis index (WOMAC) was used, validated and standardized for the patients' native language. 12 13 This questionnaire contains 17 questions regarding the level of difficulty in performing daily life activities, pain, and stiffness, to assess patient functionality. The higher the score, the worse the function. 14 In this questionnaire, a clinically relevant difference criterion is used, a possible reduction of 16% of the total score acquired before the intervention. 15 Patients were evaluated before the procedure, after 1, 3, and 6 months of the infiltrative act. The Kellgren and Lawrence (KL) classification was used in the pre-infiltration evaluation. 9

### Data Analysis Methodology

Categorical and numerical variables were tabulated and analyzed using the R (R Foundation for Statistical Computing, Vienna, Austria) software for Mac OS X, which provided measures of central tendency, percentile values, and dispersion.

To evaluate the efficacy of comparative treatments between the use of BMA and the standard treatment with articular corticosteroid infiltration and genicular block, nonparametric statistical tests were used, given the distribution of the data. The Mann-Whitney U test was applied to compare the WOMAC scores (pain, stiffness, and physical activity) between the two groups at each of the four evaluation times (pre-intervention, and at 1, 3, and 6 months posttreatment). This test was chosen because it does not assume a normal distribution of data and is suitable for independent samples.

To compare the WOMAC scores (pain, stiffness, and physical activity) for each patient before the procedure and after 6 months, the Wilcoxon signed-rank test was used, suitable for paired nonparametric samples.

To investigate the relationship between age and WOMAC scores, Spearman's correlation was used. The chi-squared test was applied to examine the relationships between gender and laterality with the scores categorized into high and low pain, stiffness, and physical activity.

Analyses were considered statistically significant with a 95% CI and a *p* -value lower than 0.05.

## Results

After 6 months of follow-up, we concluded with 35 patients, 17 in Group 1 (intervention with autologous BMA) and 18 in Group 2 (control with articular corticosteroid infiltration and genicular block). The average age of participants was approximately 58 years, with 58.06 in Group 1 and 57.78 in Group 2. Regarding age, laterality, and gender, no statistically significant differences were observed between groups 1 and 2. In terms of gender distribution, Group 1 consisted of 12 women and 5 men, while Group 2 had 13 women and 5 men.

**Fig. 7:**
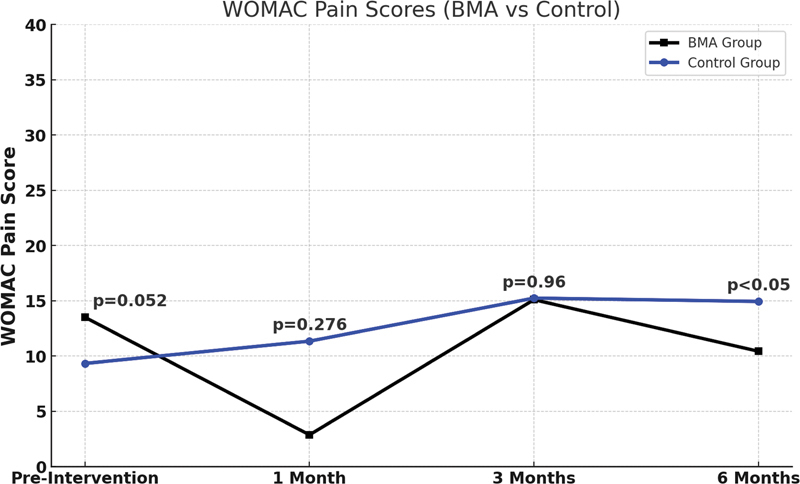
WOMAC – Pain (BMA vs. Control). Scores between the two groups at each of the four evaluation times (preintervention, and at 1, 3, and 6-months posttreatment). This subsection assesses the intensity of pain during various activities, such as walking, climbing stairs, lying down, and standing. The higher the score, the greater the severity of symptoms and patients' functional limitation. The *p* -value corresponds to the independent *t* -test between the two groups at each of the four evaluation times.

Regarding WOMAC – Pain, comparing groups 1 and 2 at the preblock stage, there was no statistically significant difference between the groups ( *p* = 0.052). At the 1 and 3-month follow-ups, the differences were also not significant ( *p* = 0.276 and 0.960, respectively). At 6 months, there was a significant difference, with the BMA group showing a lower pain score compared with the control group ( *p* = 0.030), as shown in Fig. 7 .

Regarding WOMAC – Stiffness, comparing groups 1 and 2, no statistically significant differences were observed at any of the evaluated periods ( *p* = 0.627, 0.789, and 0.097 at 1, 3, and 6 months, respectively).

Regarding WOMAC – Physical Activity, comparing groups 1 and 2, no statistically significant differences were observed at any of the evaluated periods ( *p* = 0.894, 0.960, and 0.114, at 1, 3, and 6 months, respectively).

**Fig. 8:**
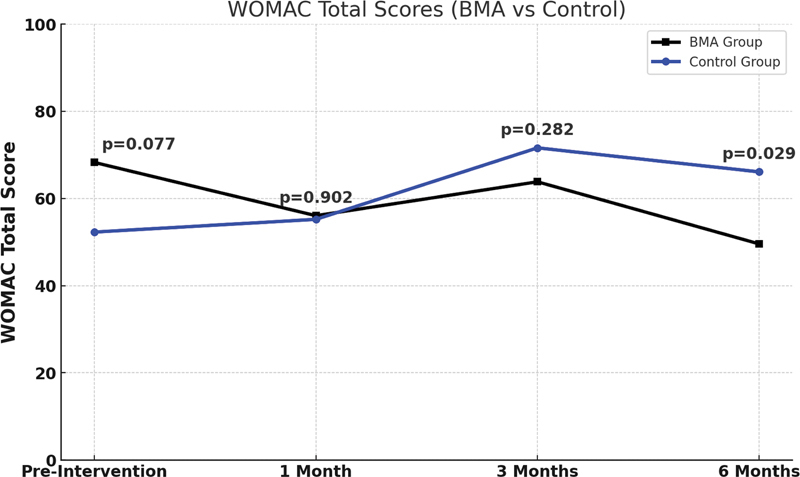
WOMAC – Total Scores (BMA vs. Control). The total scores between the two groups at each of the four evaluation times (preintervention, and at 1, 3, and 6 months posttreatment). The total score is the sum of the scores from the three subscales (Pain, Stiffness, and Physical Function). The higher the score, the greater the severity of symptoms and patients' functional limitation. The *p* -value corresponds to the independent *t* -test between the two groups at each of the four evaluation times.

However, comparing the total score over 6 months, Group 1 (BMA) showed better results. Nevertheless, although there is a trend toward a difference, it is not statistically significant at the 5% significance level ( *p* > 0.05), as shown in Fig. 8 .

The correlation between age and stiffness at 6 months was moderately positive and statistically significant (ρ = 0.366, *p* = 0.031), suggesting that older patients may experience greater stiffness over time, regardless of which technique was used. No significant associations were found between gender or laterality and WOMAC scores in any of the evaluated periods.

These results indicate that BMA treatment may offer significant benefits in reducing pain in patients with knee OA, compared with the standard treatment of articular corticosteroid infiltration and genicular block. However, despite both treatments showing improvement in physical activity and stiffness scores over time, there were no significant differences between the groups in these scores ( Table 1 ).

**Table 1:** The WOMAC scores in groups 1 (BMA) and 2 (control)

**Group 1 (BMA)**	**Preintervention**			**1-month postintervention**		
**Patient**	**Pain**	**Stiffness**	**Physical functional**	**Pain**	**Stiffness**	**Physical functional**
1	5.2	2.08	16.6	4.1	1.04	51.04
2	11.4	8.03	35	11.4	5.2	45.8
3	17.7	3.12	70.8	3.1	1.04	8.3
4	10.4	2.08	15.6	1.04	0	52.08
5	10.4	2.8	44.7	3.1	0	8.3
6	11.4	8.3	52.2	13.5	3.1	68.7
7	17.2	6.25	36.4	10.4	2.08	51.04
8	9.3	7.2	50	11.4	5.2	45.8
9	14.5	3.1	55.2	11.4	6.2	50
10	9.3	2.08	40.6	2.08	0	30.2
11	17.7	6.2	66.6	13.5	5.2	52.08
12	20.8	8.3	93.7	4.1	6.2	8.3
13	20.8	8.3	68.75	3.1	1.04	51.04
14	17.2	8.08	40.6	4.1	0	12.5
15	11.4	6.25	44.7	13.5	0	8.3
16	10.4	8.3	66.6	27	6.25	58.3
17	14.5	6.25	36.4	13.5	6.2	45.8
**Group 1 (BMA)**	**3-months postintervention**			**6-months postintervention**		
**Patient**	**Pain**	**Stiffness**	**Physical functional**	**Pain**	**Stiffness**	**Physical functional**
1	9.3	6.2	15.6	3.1	1.04	8.3
2	17.2	2.08	36.4	11.4	1.04	30.2
3	20.8	8.08	40.6	13.5	3.1	45.8
4	17.2	8.08	66.6	3.1	0	68.7
5	17.7	2.8	44.7	11.4	5.2	58.3
6	11.4	8.3	52.2	4.1	1.04	30.2
7	17.2	6.25	36.4	13.5	5.2	45.8
8	9.3	7.2	50	27	5.2	51.4
9	14.5	3.1	55.2	13.5	1.04	52.05
10	9.3	2.08	40.6	3.1	0	8.3
11	17.7	6.2	66.6	27	3.1	45.8
12	20.8	8.3	93.7	11.4	6.2	8.3
13	20.8	8.3	68.75	3.1	6.2	51.04
14	17.2	8.08	40.6	4.1	0	12.5
15	11.4	6.25	44.7	13.5	1.4	8.3
16	10.4	8.3	66.6	11.4	6.25	58.3
17	14.5	6.25	36.4	3.1	6.2	30.2
**Group 2 (control)**	**Preintervention**			**1-month postintervention**		
**Patient**	**Pain**	**Stiffness**	**Physical functional**	**Pain**	**Stiffness**	**Physical functional**
1	4.1	1.04	51.04	9.3	0	52.08
2	11.4	5.2	45.8	10.4	3.1	51.04
3	3.1	1.04	8.3	11.4	2.08	30.2
4	1.04	0	52.08	3.1	5.2	45.8
5	3.1	0	8.3	4.1	1.04	30.8
6	13.5	3.1	68.7	13.5	6.2	68.7
7	10.4	2.08	51.04	10.4	2.08	52.08
8	11.4	5.2	45.8	13.5	2.08	45.8
9	11.4	6.2	50	11.4	6.2	30.2
10	2.08	0	30.2	27	1.04	45.8
11	13.5	5.2	52.08	13.5	5.2	52.08
12	4.1	6.2	8.3	4.1	6.2	8.3
13	3.1	1.04	51.04	3.1	1.04	51.04
14	4.1	0	12.5	4.1	0	12.5
15	13.5	0	8.3	13.5	0	30.2
16	27	6.25	58.3	27	6.25	51.04
17	13.5	6.2	45.8	13.5	6.2	30.2
18	17.7	8.3	68.75	11.4	2.08	45.8
**Group 2 (control)**	**3-months postintervention**			**6-months postintervention**		
**Patient**	**Pain**	**Stiffness**	**Physical functional**	**Pain**	**Stiffness**	**Physical functional**
1	13.5	6.2	15.6	20.8	6.25	45.8
2	17.2	2.08	36.4	4.1	3.1	30.2
3	20.8	8.08	40.6	13.5	3.1	52.05
4	17.2	8.08	66.6	20.8	5.2	68.7
5	17.7	2.8	44.7	11.4	5.2	58.3
6	11.4	8.3	52.2	4.1	6.25	51.4
7	17.2	6.25	36.4	13.5	5.2	45.8
8	9.3	7.2	50	27	5.2	51.4
9	14.5	3.1	55.2	13.5	1.04	52.05
10	9.3	2.08	40.6	13.5	3.1	30.2
11	17.7	6.2	66.6	27	5.2	45.8
12	20.8	8.3	93.7	11.4	6.2	68.75
13	20.8	8.3	68.75	17.7	6.2	51.04
14	17.2	8.08	40.6	13.5	1.4	12.5
15	11.4	6.25	44.7	13.5	3.1	30.2
16	10.4	8.3	66.6	11.4	5.2	58.3
17	14.5	6.25	36.4	20.8	6.2	30.2
18	13.5	1.04	52.2	11.4	3.1	58.3

**Abbreviation:** BMA, bone marrow aspirate; WOMAC, Western Ontario and McMaster universities osteoarthritis index.

## Discussion

This study evaluated the efficacy of treating knee OA using autologous BMA compared with joint corticosteroid infiltration and genicular block. Regardless of the group, all patients showed clinical improvement. However, results after 6 months of follow-up indicate a significant reduction in pain for the group treated with BMA, suggesting an improvement in the quality of life for these patients.

The BMA technique has been highlighted in various orthopedic conditions due to its regenerative potential and associated low risk. Specifically, BMA provides a rich supply of regenerative cells capable of differentiating into various tissue types, representing a promising approach for treating OA. With the current understanding of the inflammatory mechanisms in OA, BMA is considered a relevant therapeutic alternative. 5 7 This treatment is based on MSCs, which are pluripotent, meaning they have the ability to differentiate into a variety of tissues, including osteocytes, chondrocytes, adipocytes, mast cells, fibroblasts, and hematopoietic precursor cells. 16 Moreover, these cells have immunomodulatory properties and can suppress chondrocyte apoptosis. 17

One of the advantages of BMA as a source of MSCs is that its technique is relatively simple. It is a percutaneous procedure with low morbidity because it is an autologous source and does not require processing, unlike bone marrow aspirate concentrate (BMAC) and adipose sources, which need several processing steps. 5 18 Regarding standardization, there is no universally standardized protocol for processing. Different clinics and researchers may use slightly different parameters. The efficacy of BMAC may depend on the quality and purity of the preparation. As for regulations and guidelines, depending on the country or region, there may be specific regulations governing its processing and use. In some countries, the use of processed bone marrow is only allowed through research protocols.

The preferred site for obtaining the aspirate is usually the posterior iliac crest, a safe location that presents fewer complications and has a higher quantity of MSCs compared with the anterior iliac crest. 19 However, there is still no consensus on some aspects of the technique, such as patient positioning, anesthesia, and the choice of collection needles. 20 21 The main factor of the technique is to maintain constant and low aspiration pressure, choosing syringes of 5 or 10 ml. 22 This is because MSCs are diluted in blood when aspirated, causing 85% of the available cells to be collected in the first 2 ml of aspirate. Therefore, every 2 ml collected, the needle should advance 0.5 to 1 cm to optimize collection. 5

Bastos et al. 23 studied the efficacy and safety of intraarticular injections of expanded autologous stromal MSCs obtained from bone marrow aspiration (±80–100 ml) from both posterior iliac crests in patients with knee OA. These authors concluded that intraarticular injections of expanded MSCs alone or in combination with PRP are safe and have a beneficial effect on symptoms in patients with symptomatic knee OA. These patients were evaluated with a functionality and quality of life questionnaire called the knee and osteoarthritis outcome score (KOOS) at intervals of 1, 2, 3, 6, 9, and 12 months. Similar to the other study, an initial improvement peak was observed in the first 2 months, stabilizing until the 9 ^th^ month and then experiencing a slight improvement in the 12 ^th^ month.

Garay-Mendoza et al. 24 conducted a prospective, open-label, phase I/II clinical trial to assess the safety and efficacy of a single intraarticular injection of autologously stimulated bone marrow stem cells (BM-SC) in patients with knee OA. The BM-SC were obtained by aspiration and administered in a single intraarticular injection. The control group received only oral paracetamol. The visual analog scale (VAS) and WOMAC scores were obtained at 1-week, 1-month, and 6-months in both groups. Patients showed significant improvement, especially in VAS, which was superior to the control group. This study demonstrated the viability and efficacy of a procedure that can be performed on an outpatient basis for treating knee OA.

Despite promising results, the long-term efficacy and optimization of BMA treatment protocols still require validation through additional research. The regenerative potential of MSCs, although promising, is not yet fully understood. Larger and more rigorous studies are needed to consolidate BMA as a viable and effective treatment option for knee OA. 7 In our study, the absence of significant differences between groups in terms of age and gender suggests that it may be applicable to a wide range of patients without the need for selection based on these demographic criteria. This is encouraging, as it indicates that the benefits of the treatment can be generalized across both genders and a wide age range.

In this context, as regenerative medicine advances, the use of BMA for knee OA remains in an exciting phase of development. Enhanced understanding of how these therapies can be integrated into existing treatment plans could significantly alter the approach to managing OA, offering new hopes for reducing pain and improving the quality of life for patients.

Regarding the limitations of this study, we highlight the sample size and duration of follow-up. Unfortunately, we experienced a loss of follow-up with patients. We started the study with 50 patients, 25 in each group, and ended with only 35. Future studies should address these limitations, ideally with larger samples and longer follow-up periods, to confirm and expand the reported findings. Another limitation of the study was that we did not include important demographic data, such as body mass index and the degree of OA in each individual.

## Conclusion

Treatment with BMA can significantly reduce pain, potentially leading to improved knee functionality, suggesting its potential as a viable therapeutic option for managing knee OA.
